# Supplementary material for: Phenotypic Differentiation Between Highland and Coastal Quinoa Under Cold Stress Conditions
Source: Plant Cell Environ. 2026 Jul 5;49(11):7976–96. doi: 10.1111/pce.70707 (PMC13646688; doi:10.1111/pce.70707)
Supplement: Supplementary file 3 [file PCE-49-7976-s004.pdf]

Supplementary tables

Table S1. Details of sowing and harvesting of the three different time points.

Time point	Date of sowing	Seeds per hill	Plants per plot	Date of final harvesting
S1	04 March	1	10	31st October
S2	01 April	1	10	31st October
S3	30 April	3	30	31st October

Table S2. Weather conditions during the crop growth period

Month	Relative humidity (%)		Monthly soil temperature (°C)		Monthly air temperature (°C)		Frost days*	
	Mean	Min.	Max.	Mean	Min.	Max.	Mean	
March	71.2	2.7	11.9	5.6	-5.4	24.5	5.0	14
April	65.0	4.3	12.4	8.3	-4.3	23.6	6.9	11
May	72.2	8.1	14.5	11.6	-1.4	30.7	10.9	1
June	74.0	12.3	20.8	17.6	4.1	33.8	18.9	0
July	79.5	16.6	22.4	19.2	8.7	30.3	18.4	0
August	82.3	15.7	21.1	18.4	6.8	33.6	16.8	0
September	81.0	13.1	18.6	16.3	2.2	29.6	15.3	0
October	86.2	7.4	15.2	11.1	-2.8	22.6	8.4	4

†Frost days represents the number of days when the minimum air temperature is below 0°C.

Table S3. mAP@[.5:.95] and penalty result of models with different hyperparamers. Each unique hyperparameter combination (models 1-12) was replicated five times (A-E).

Model	Loss	Heads.m	Mask_resolution	mAP@[.5:.95]	Penalty
1-A	mask1class1	heads	28x28	58.47	-
1-B	mask1class1	heads	28x28	54.60	-
1-C	mask1class1	heads	28x28	54.99	-
1-D	mask1class1	heads	28x28	51.62	-
1-E	mask1class1	heads	28x28	52.33	-
2-A	mask1class10	heads	28x28	60.26	-
2-B	mask1class10	heads	28x28	61.69	-
2-C	mask1class10	heads	28x28	60.26	-
2-D	mask1class10	heads	28x28	55.07	-
2-E	mask1class10	heads	28x28	55.63	-
3-A	mask10class1	heads	28x28	57.38	-
3-B	mask10class1	heads	28x28	55.53	-
3-C	mask10class1	heads	28x28	51.55	-
3-D	mask10class1	heads	28x28	57.22	-
3-E	mask10class1	heads	28x28	57.05	-
4-A	mask1class1	heads	56x56	53.89	-
4-B	mask1class1	heads	56x56	59.08	-
4-C	mask1class1	heads	56x56	59.48	-
4-D	mask1class1	heads	56x56	58.10	-
4-E	mask1class1	heads	56x56	57.12	57
5-A	mask1class10	heads	56x56	57.63	-

5-B	mask1class10	heads	56x56	62.01	-
5-C	mask1class10	heads	56x56	60.80	-
5-D	mask1class10	heads	56x56	55.19	-
5-E	mask1class10	heads	56x56	56.76	-
6-A	mask10class1	heads	56x56	58.37	-
6-B	mask10class1	heads	56x56	59.36	-
6-C	mask10class1	heads	56x56	54.03	-
6-D	mask10class1	heads	56x56	58.73	-
6-E	mask10class1	heads	56x56	54.64	-
7-A	mask1class1	all	28x28	55.55	-
7-B	mask1class1	all	28x28	53.02	-
7-C	mask1class1	all	28x28	56.75	-
7-D	mask1class1	all	28x28	58.34	-
7-E	mask1class1	all	28x28	55.21	-
8-A	mask1class10	all	28x28	54.49	-
8-B	mask1class10	all	28x28	63.35	23
8-C	mask1class10	all	28x28	63.16	22
8-D	mask1class10	all	28x28	59.58	-
8-E	mask1class10	all	28x28	57.20	-
9-A	mask10class1	all	28x28	59.66	-
9-B	mask10class1	all	28x28	52.96	-
9-C	mask10class1	all	28x28	60.31	-
9-D	mask10class1	all	28x28	62.95	-
9-E	mask10class1	all	28x28	54.81	-

10-A	mask1class1	all	56x56	58.73	-
10-B	mask1class1	all	56x56	59.39	-
10-C	mask1class1	all	56x56	61.25	-
10-D	mask1class1	all	56x56	62.88	-
10-E	mask1class1	all	56x56	58.62	-
11-A	mask1class10	all	56x56	61.86	-
11-B	mask1class10	all	56x56	58.38	-
11-C	mask1class10	all	56x56	62.84	-
11-D	mask1class10	all	56x56	64.39	24
11-E	mask1class10	all	56x56	64.38	24
12-A	mask10class1	all	56x56	53.94	-
12-B	mask10class1	all	56x56	58.37	-
12-C	mask10class1	all	56x56	55.47	-
12-D	mask10class1	all	56x56	49.78	-
12-E	mask10class1	all	56x56	59.47	-

Table S4. Coefficient of vectors on the principal components

Traits	PC 1	PC 2	PC 3	PC 4
MET	0.289	0.401	-0.487	-0.178
EP	-0.146	-0.160	0.687	0.527
DTP	0.817	0.101	-0.409	0.283
DTF	0.745	0.460	-0.131	0.283
DTM	-0.388	0.775	0.270	0.199
PH	0.809	0.261	0.386	0.0594
PL	0.195	0.553	0.562	-0.517
PW	0.645	-0.363	0.406	-0.167
SY	0.703	-0.498	0.133	-0.106

†Note: Mean emergence time (MET), emergence percentage (EP), days to panicle emergence (DTP), days to flowering (DTF), days to maturity (DTM), plant height (PH), panicle length (PL), panicle width (PW), seed yield (SY).

Table S5. Estimates of the original mean (Xo), the mean of the selected individuals (Xs), and the selection differential (SD).

**Trait**	**X_o**	**X_s**	**SD**
MET	17.8	17.2	-0.521
EP	33.3	34.2	0.843
DTF	97.4	94.9	-2.47
DTM	189	167	-21.8
PH	158	157	-1.31
PW	6.95	8.29	1.34
SY	48.5	70.7	22.2

†Note: Mean emergence time (MET), emergence percentage (EP), days to flowering (DTF), days to maturity (DTM), plant height (PH), panicle width (PW), seed yield (SY).

Table S6. Estimated means (BLUEs) of the top eight genotypes selected by multi-trait stability index for their stability in three sowing dates (S1, S2, and S3) for the traits Mean emergence time (MET), emergence percentage (EP), days to flowering (DTF), days to maturity (DTM), plant height (PH), panicle width (PW) and seed yield (SY)

Time point	Accession	MET	EP	DTF	DTM	PH	PW	SY
S1	Titicaca	25.9	43.3	102.1	160.2	117.56	11.12	78.92
S1	PI-614886	27.1	23.3	110.4	160.4	146.0	6.35	220.2
S1	PI-614882	26.10	36.9	130.3	174.43	202.7	10.8	90.31
S1	PI-634923	26.2	14.3	112.3	159.0	165.3	8.8	89.8
S1	CHEN-160	26	26.6	113.3	216	121.7	8.3	58.6
S1	CHEN-465	26.1	23.8	109.9	217.5	172.2	6.6	71.9
S1	CHEN-69	26.4	33.3	126	232	161.8	8.25	73.7
S1	CHEN-91	26.3	36.6	126.6	229	201.8	9.3	74.5
S2	Titicaca	14.9	50	78	132	115.1	9.25	49.6
S2	PI-614886	13.01	37.76	90.2	132.8	147.3	9.11	158.9
S2	PI-614882	15.9	40	103.3	148.6	201	8.63	88.5
S2	PI-634923	15.3	20	94.6	134	136.8	6.5	42.3
S2	CHEN-160	15.2	23.3	94.6	187	144.8	7.5	45.45
S2	CHEN-465	15.09	14.7	92.0	190.8	157.1	5.01	44.6
S2	CHEN-69	15.7	32.5	101.4	203.8	166.8	6.8	46.7
S2	CHEN-91	14	40	104	207	199.9	9.9	61.4
S3	Titicaca	10.3	36.6	59.6	106	101.2	7	31.3
S3	PI-614886	10.19	33.3	69	113	155.3	10.76	110.49
S3	PI-614882	10.1	43.3	78	122.6	181.05	7.16	51.6
S3	PI-634923	11.9	21.2	72.6	107.4	144.5	8.94	47.6

S3	CHEN-160	10.6	43.3	77	160.3	131.7	7.3	34.4
S3	CHEN-465	10.3	54.5	71.8	166.0	139.7	6	34.2
S3	CHEN-69	11.05	36.6	81.6	181	160	9.7	47.4
S3	CHEN-91	9.45	50	82.6	181.3	178.3	9.08	53.8

Table S7. Wald F-test results for fixed effects in quinoa cold stress laboratory experiment, reported as p-values

		Source of variation			
**Method**	**Traits**	**Rep**	**Treatment**	**ID**	**ID:Treatment**
Manual documentation	MGT	0.202	0 ****	0 ****	0 ****
	GP	0.737	0.567	0 ****	0 ****
	VGP	0.306	0.002 **	0 ****	0.001 ***
Image analysis	MGT	0.007**	0 ****	0 ****	0 ****
	GP	0.434	0.423	0 ****	0.003**
	Growth Rate	0 ****	0 ****	0 ****	0 ****

†Note: Mean germination time (MGT), germination percentage (GP), viable germination percentage (VGP). Asterisks (*p < 0.05, **p < 0.01, ***p < 0.001, ****p < 0.0001).

Table S8. Least squares means of mAP values of significant model parameters. Means with the same letter are not significantly different.

Parameter	p-value	Least squares means	Standard error
**heads.m**	0.048222 *		
all		58.57[a]	0.56
heads		56.96[b]	0.56
**loss**	0.003501 **		
mask1class10		59.75[a]	0.69
mask1class1		56.97[b]	0.69
mask10class1		56.58[b]	0.69

Table S9. Result of penalty scoring of the validation images from another germination experiment used for evaluation of the pipeline.

Image Set	Model Update	Seed/ seedlings error	Missed Seed/ seedlings	Multiple detections of same seed/ seedling	Multiple seed/ seedlings detected as one	Other update	Total Penalty count
All images (207 seeds)	without update	0	203	0	2	13	218
	10 epochs, replicate 1	12	117	5	7	3	144
	10 epochs, replicate 2	20	106	1	5	0	132
	10 epochs, replicate 3	8	138	1	10	0	157
	30 epochs, replicate 1	14	96	9	21	5	145
	30 epochs, replicate 2	11	79	4	13	3	110
	30 epochs, replicate 3	12	83	3	11	3	112
	50 epochs, replicate 1	9	86	3	9	3	110
	50 epochs, replicate 2	51	109	0	1	2	163
	50 epochs, replicate 3	20	127	10	21	9	187

Images with 10-30 seeds (157 seeds)	without update	0	156	0	1	6	163
	10 epochs, replicate 1	9	108	0	6	3	126
	10 epochs, replicate 2	14	95	0	4	0	113
	10 epochs, replicate 3	5	127	0	8	0	140
	30 epochs, replicate 1	10	91	2	20	1	124
	30 epochs, replicate 2	9	77	0	12	2	100
	30 epochs, replicate 3	9	82	1	10	1	103
	50 epochs, replicate 1	8	84	0	8	1	101
	50 epochs, replicate 2	26	101	0	1	0	128
	50 epochs, replicate 3	12	116	4	18	7	157
Images with 5 seeds (50 seeds)	without update	0	47	0	1	7	55
	10 epochs, replicate 1	3	9	5	1	0	18
	10 epochs, replicate 2	6	11	1	1	0	19
	10 epochs, replicate 3	3	11	1	2	0	17
	30 epochs, replicate 1	4	5	7	1	4	21

	30 epochs, replicate 2	2	2	4	1	1	10
	30 epochs, replicate 3	3	1	2	1	2	9
	50 epochs, replicate 1	1	2	3	1	2	9
	50 epochs, replicate 2	25	8	0	0	2	35

†1: Other errors included for example when part of the labelling or the Petri dish was detected as seedling or when a seed was detected as QR-code.

Table S10. Result of penalty scoring of the random images from another germination experiment analyzed with the best updated model (30 epochs, replicate 2).

Image set	All 20 images (191 seeds)	Images with 10+ seeds (141 seeds)	Images with 5 seeds (50 seeds)
**Seed/seedling error**	16	11	5
**Missed seeds/seedlings**	77	71	6
**Multiple detections of the same seed/seedling**	3	1	2
**Multiple seeds/seedlings detected as one**	11	11	0
**Other errors**[1]	3	1	2
**Total penalty count**	110	95	15

†1: Other errors included for example when part of the labelling or the Petri dish was detected as seedling or when a seed was detected as QR-code.
